# Clinically complete spinal cord injury is not always anatomically complete

**DOI:** 10.3389/fneur.2026.1882708

**Published:** 2026-07-30

**Authors:** Nicolas Serratrice

**Affiliations:** 1 NeuroFAT, Marseille, France; 2 Institut Marseille Rachis, Vert Coteau Private Hospital, Marseille, France; 3 Laboratory of Anatomy, Aix-Marseille University, Marseille, France

**Keywords:** ASIA impairment scale, clinically complete SCI, imaging biomarkers, magnetic resonance imaging, neurological prognosis, spinal cord contusion, spinal cord hemorrhage, spinal cord injury

## Abstract

**Background:**

The classification of traumatic spinal cord injury (SCI) traditionally relies on the distinction between complete and incomplete neurological deficits using the American Spinal Injury Association (ASIA) Impairment Scale (AIS). However, the relationship between clinical completeness and underlying anatomical integrity remains complex and frequently misunderstood.

**Objective:**

To revisit the anatomical reality of clinically complete SCI, analyze lesion patterns observed on magnetic resonance imaging (MRI), and discuss the clinical and therapeutic implications of the clinico-radiological dissociation, with particular emphasis on anatomically informed perspectives.

**Methods:**

This narrative review synthesizes classical and contemporary literature addressing the pathological substrates of traumatic SCI, MRI-based lesion characterization, and correlations with neurological outcomes.

**Results:**

Clinically complete SCI (AIS A) does not invariably correspond to anatomical transection. MRI studies consistently demonstrate heterogeneous lesion morphologies, including cord edema, contusion, intramedullary hemorrhage, compression, and, less frequently, transection. Intramedullary hemorrhage and extensive cord damage are strongly associated with severe neurological deficits and poorer recovery, whereas edema-dominant patterns may reflect partially preserved tissue integrity. These observations highlight the limitations of relying exclusively on neurological classification to infer structural severity and support the integration of anatomical MRI biomarkers into clinically meaningful prognostic frameworks.

**Conclusion:**

Recognizing the discrepancy between clinical presentation and anatomical reality refines prognostic interpretation and supports a multidimensional assessment integrating neurological examination with advanced imaging. This perspective carries important implications for surgical decision-making, neuroprotective strategies, emerging therapies, and rehabilitation pathways.

## Graphical abstract

**Figure:**
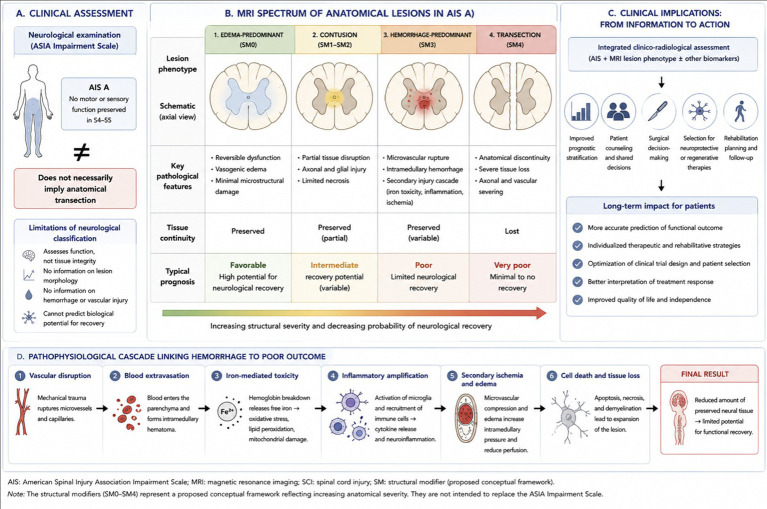

## Introduction

Traumatic spinal cord injury (SCI) remains one of the most devastating conditions encountered in neurotrauma, leading to profound motor, sensory, and autonomic impairments with major long-term functional consequences (1). Despite advances in early surgical management and intensive rehabilitation strategies, neurological recovery remains highly variable and difficult to predict.

The clinical assessment of injury severity primarily relies on the American Spinal Injury Association (ASIA) Impairment Scale (AIS), which distinguishes between complete (AIS A) and incomplete injuries (AIS B–E) based on the preservation of motor and sensory function below the neurological level of injury (2). This classification has become a cornerstone of clinical practice, prognostic evaluation, and research standardization in SCI (3).

However, the concept of a “complete” SCI remains frequently misunderstood. A clinically complete injury (AIS A), defined by the absence of detectable motor and sensory function below the lesion, does not necessarily imply a complete anatomical transection of the spinal cord. Experimental and clinical observations have consistently demonstrated that many patients classified as AIS A may present with preserved tissue continuity on imaging, typically characterized by patterns of edema, contusion, or intramedullary hemorrhage rather than frank transection (4, 5).

Magnetic resonance imaging (MRI) has emerged as a critical tool for evaluating the structural integrity of the injured spinal cord. MRI allows characterization of lesion morphology, including cord edema, contusion, hemorrhage, compression, and transection, each of which carries distinct prognostic implications (6). Notably, intramedullary hemorrhage and extensive cord damage have been associated with more severe neurological deficits and poorer functional recovery, whereas edema-dominant patterns may retain a greater potential for neurological improvement (6, 7).

This distinction is not merely conceptual but directly impacts prognosis and therapeutic reasoning. Overreliance on the clinical concept of injury completeness may obscure the heterogeneity of underlying pathological mechanisms, influence therapeutic decision-making, and potentially limit consideration of neuroprotective or regenerative strategies. A refined understanding of the anatomical substrates of neurologically complete injuries is therefore essential for improving prognostic accuracy and guiding emerging therapeutic approaches.

The aim of this review is to revisit the anatomical reality of clinically complete SCI, analyze the spectrum of lesion patterns observed on MRI, and discuss the clinical and therapeutic implications of this clinico-radiological dissociation, with particular emphasis on anatomically informed perspectives.

## Review

### Limitations of clinical classification alone

The AIS has become the universal standard for neurological assessment in SCI. Its widespread adoption has significantly improved communication between clinicians, facilitated prognostic stratification, and standardized research reporting. Nevertheless, the AIS classification remains fundamentally a functional clinical tool, rather than a direct reflection of the underlying anatomical integrity of the spinal cord.

A clinically complete SCI (AIS A) is defined by the absence of motor and sensory function below the neurological level of injury, including sacral segments. While this definition is clinically robust and reproducible, it does not provide information regarding the structural preservation or destruction of spinal cord tissue. In clinical practice, neurological examination captures the functional consequences of the injury but cannot fully characterize the heterogeneous pathological substrates responsible for these deficits.

Several studies have demonstrated that neurological completeness does not systematically correspond to anatomical transection. Early MRI investigations revealed that many patients classified as AIS A present with preserved spinal cord continuity, most commonly associated with patterns of cord edema, contusion, or intramedullary hemorrhage rather than frank transection (4, 5). These observations highlight that functional loss may result from complex mechanisms including axonal disruption, microvascular injury, ischemia, and secondary inflammatory cascades, even in the absence of macroscopic tissue discontinuity.

Furthermore, neurological assessment in the acute phase is influenced by multiple factors. Spinal shock, evolving secondary injury processes, sedation, associated traumatic brain injury, and patient cooperation may all affect the reliability of early classification. Consequently, an initial AIS A designation does not invariably predict permanent neurological absence, as partial recovery may occur in a subset of patients (3).

This clinico-anatomical dissociation underscores an important limitation of relying exclusively on neurological classification. While AIS grading remains indispensable for clinical evaluation, it should not be interpreted as a surrogate marker of anatomical destruction. A comprehensive assessment of SCI severity therefore requires integration of clinical findings with advanced imaging modalities capable of characterizing lesion morphology and extent.

### Anatomical patterns of SCI

Traumatic SCI encompasses a broad spectrum of pathological mechanisms and lesion morphologies, reflecting the complex biomechanical forces applied to the spinal column and neural elements. Rather than representing a binary phenomenon, spinal cord damage occurs along a continuum ranging from subtle microstructural alterations to extensive tissue destruction. These lesion morphologies reflect complex primary and secondary injury mechanisms (8).

At the milder end of the spectrum, SCI may primarily manifest as cord edema, resulting from vascular permeability changes, ionic imbalance, and early inflammatory responses. Edema represents potentially reversible tissue dysfunction and is frequently associated with incomplete neurological deficits. Although the clinical presentation may be severe, tissue continuity is typically preserved.

Spinal cord contusion constitutes an intermediate stage of injury, characterized by focal parenchymal damage involving axonal disruption, microhemorrhages, and varying degrees of necrosis. Contusions are among the most common anatomical substrates observed in traumatic SCI and reflect the combined effects of direct mechanical insult and secondary injury cascades. Neurological outcomes in contusive injuries are highly variable, depending on lesion extent and the presence of associated hemorrhagic components. Although cord contusion is predominantly associated with incomplete neurological deficits, clinically complete presentations may also occur. In such cases, severe functional impairment may arise from complex microstructural and vascular alterations rather than macroscopic tissue discontinuity.

Intramedullary hemorrhage represents a critical pathological event associated with more severe neurological impairment. Hemorrhagic lesions indicate disruption of the spinal cord microvasculature, leading to direct cellular toxicity, increased local pressure, ischemia, and expansion of secondary injury. Numerous studies have demonstrated that the presence and longitudinal extent of hemorrhage correlate strongly with poorer neurological recovery (4, 6, 7).

In many clinical scenarios, SCI is compounded by extrinsic compression, resulting from fracture fragments, dislocation, ligamentous instability, epidural hematoma, or disc herniation. Compression contributes to ischemia and exacerbates secondary injury mechanisms. Importantly, decompressive surgery may modify the evolution of these lesions, underscoring the dynamic nature of spinal cord pathology in the acute phase.

At the most severe end of the spectrum lies spinal cord transection, defined by macroscopic anatomical discontinuity of neural tissue. True transection remains relatively uncommon in clinical practice and is typically associated with high-energy trauma and profound neurological deficits. Even in these extreme cases, partial tissue bridges may occasionally persist, illustrating the complexity of lesion characterization.

Traumatic SCI distribution also varies according to the anatomical level involved. Epidemiological studies consistently report that cervical injuries represent the most frequent localization, accounting for approximately half of traumatic spinal cord injuries, followed by thoracic and, less commonly, lumbar lesions. This predominance reflects both the biomechanical vulnerability and mobility of the cervical spine. Thoracic injuries, often associated with high-energy trauma, may result in severe neurological deficits due to the relative narrowness of the spinal canal. Lumbar spinal cord involvement is less common given the transition to *cauda equina* structures, which present distinct pathophysiological and clinical characteristics (3, 9).

These diverse anatomical patterns illustrate that traumatic SCI is not a uniform entity. Distinct lesion morphologies reflect different pathophysiological processes and carry markedly different implications for neurological prognosis, therapeutic strategies, and potential for recovery.

### Role of MRI in lesion characterization and prognosis (Table 1)

**Table 1:** MRI lesion patterns and associated prognostic implications reported in representative acute SCI studies.

Code	Anatomical Pattern	Physiopathology dominante
SM0	No visible cord abnormality	Dysfonction microscopique
SM1	Predominant edema	Dysfonction potentiellement réversible
SM2	Contusion / mixed lesion	Lésion structurale partielle
SM3	Intramedullary hemorrhage	Lésion sévère / pronostic défavorable
SM4	Anatomical transection	Destruction majeure

MRI has emerged as a key diagnostic and prognostic tool in acute SCI (10). MRI has fundamentally transformed the evaluation of traumatic SCI by enabling direct visualization of the injured neural tissue. Unlike clinical examination alone, MRI provides critical insights into lesion morphology, extent, and structural integrity, thereby refining both diagnostic assessment and prognostic estimation. Current clinical practice guidelines emphasize the central role of MRI in acute SCI evaluation (11).

Early MRI studies established that SCI encompasses a heterogeneous spectrum of imaging patterns. Kulkarni *et al.* first demonstrated that acute SCI could be characterized by distinct signal abnormalities reflecting edema, contusion, hemorrhage, or transection, each associated with different clinical outcomes (4). Subsequent investigations further clarified the prognostic significance of these lesion types.

Ramón et al. provided one of the seminal clinico-radiological correlations in acute SCI, classifying injuries into three principal MRI patterns: Type I (intramedullary hemorrhage), Type II (cord edema), and Type III (cord contusion), each associated with distinct neurological profiles and outcomes. In their retrospective series of 55 patients, 28 individuals (51%) were classified as clinically complete injuries (AIS A) at admission. MRI findings revealed marked differences in lesion morphology. Intramedullary hemorrhage (Type I lesions) was observed in 15 cases, all of which (100%) corresponded to clinically complete injuries. In contrast, edema-dominant lesions (Type II) were predominantly associated with incomplete injuries, with approximately 93% of patients demonstrating preserved neurological function. Contusive lesions (Type III) displayed intermediate behavior, with 70% of cases classified as incomplete and 30% as complete injuries. True spinal cord transection was identified in only two patients, both presenting with clinically complete deficits (5).

More recent prospective data confirm the predominance of non-transection injury patterns. Naik *et al.*, in a cohort of 57 patients, reported that cord edema represented the most frequent MRI finding (40.4%), followed by cord contusion/edema (17.5%). Intramedullary hemorrhage was observed in 5.3% of cases, while imaging evidence of cord transection with contusion/edema accounted for only 7.0% of lesions. These results emphasize that edema, contusion, and hemorrhagic patterns constitute the majority of acute MRI presentations, whereas frank transection remains relatively rare. Notably, both transection and intramedullary hemorrhage were associated with markedly worse neurological outcomes (12).

Beyond lesion categorization, quantitative MRI parameters have demonstrated robust prognostic value across SCI subtypes (13). Miyanji *et al.*, in a prospective study of 100 patients with acute cervical SCI, showed that maximum canal compromise, the presence of intramedullary hemorrhage, and lesion length were strongly correlated with baseline neurological severity and long-term functional recovery. In particular, intramedullary hemorrhage and extensive cord swelling were consistently associated with poorer neurological prognosis (6).

Despite these advances, large national registries such as the Spinal Cord Injury Model Systems (SCIMS) and the Rick Hansen Spinal Cord Injury Registry (RHSCIR) primarily rely on neurological classification scales and functional outcomes. Although these databases contain valuable imaging data, few studies derived from registry datasets have systematically analyzed anatomical lesion patterns without centralized MRI review. Ongoing efforts to establish imaging repositories may help bridge this gap and enable large-scale characterization of spinal cord lesion morphology.

Collectively, MRI findings reinforce the concept that clinically complete SCI does not invariably correspond to anatomical transection. Instead, a spectrum of potentially distinct pathological substrates underlies severe neurological presentations, with major implications for prognosis and therapeutic strategy.

### Clinical and therapeutic implications

Recognizing the discrepancy between clinical classification and anatomical reality carries important consequences for patient management. While neurological examination remains indispensable, integrating structural information provided by MRI refines both prognostic assessment and therapeutic decision-making. Lesion morphology may also be may be particularly informative for selected therapeutic decisions as neurological grading in early therapeutic reasoning.

From a surgical perspective, lesion morphology plays a critical role. In the presence of persistent spinal cord compression, fracture-dislocation, epidural hematoma, or mechanical instability, early decompression remains a central therapeutic objective. MRI not only confirms the presence and severity of compression but also characterizes intramedullary damage. Extensive hemorrhagic lesions or imaging evidence of cord disruption are frequently associated with more severe neurological deficits and may inform discussions regarding expected recovery. Conversely, edema-dominant patterns may indicate partially preserved tissue integrity, supporting a more cautious prognostic interpretation.

Importantly, MRI-based lesion characterization challenges deterministic interpretations of clinically complete injuries. Patients initially classified as AIS A may still present with preserved spinal cord continuity, suggesting that severe neurological dysfunction may arise from reversible or evolving pathological processes, including ischemia, inflammation, and secondary injury cascades. This observation has direct implications for therapeutic windows, particularly in the context of early intervention strategies.

Beyond surgical considerations, understanding lesion morphology is fundamental to the development of neuroprotective approaches. Secondary injury mechanisms, including excitotoxicity, oxidative stress, microvascular dysfunction, and inflammatory responses, contribute significantly to tissue damage progression. Anatomical patterns observed on MRI, particularly cord edema and contusion, may reflect dynamic processes potentially amenable to targeted interventions. Conversely, extensive intramedullary hemorrhage often represents more advanced and less reversible tissue destruction.

These considerations are also highly relevant for emerging therapeutic strategies (14, 15). Regenerative and cell-based approaches, as well as pharmacological neuroprotection, increasingly rely on precise lesion characterization to identify patients with residual viable tissue and potential for recovery. The assumption that clinical completeness equates to anatomical destruction may therefore obscure therapeutic opportunities.

Finally, the clinico-anatomical dissociation holds major implications for rehabilitation. Functional recovery potential is intimately linked to preserved neural substrates. Overly pessimistic interpretations based solely on clinical completeness may influence early rehabilitation planning, patient counseling, and long-term expectations. A more nuanced understanding of lesion morphology supports individualized prognostic frameworks and integrated care pathways.

Collectively, these observations emphasize that SCI management benefits from a multidimensional assessment integrating neurological evaluation with anatomical imaging rather than reliance on a single classification paradigm. Among MRI findings, intramedullary hemorrhage has consistently emerged as the most robust predictor of poor neurological outcome, reflecting not only injury severity but also the biological processes driving secondary tissue damage.

### Intramedullary hemorrhage: the current benchmark MRI biomarker

Among all conventional MRI biomarkers, intramedullary hemorrhage has consistently emerged as the strongest predictor of poor neurological recovery following traumatic SCI. Importantly, hemorrhage should not be regarded solely as a marker of injury severity but also as an active contributor to secondary tissue damage.

Hemorrhagic lesions reflect disruption of the spinal cord microvasculature, indicating that the initial mechanical trauma has caused extensive vascular destruction in addition to axonal injury. Blood extravasation into the spinal cord parenchyma initiates a cascade of secondary injury mechanisms that amplifies tissue damage beyond the primary insult. Hemoglobin degradation releases free iron, promoting oxidative stress, lipid peroxidation, mitochondrial dysfunction, and neuronal as well as oligodendroglial cell death. Simultaneously, blood-derived products activate resident microglia, recruit peripheral inflammatory cells, and sustain a pro-inflammatory environment that contributes to lesion expansion.

Hemorrhage also increases intramedullary pressure and compromises spinal cord perfusion, creating a vicious cycle of ischemia, edema, inflammation, and apoptosis. As a consequence, the amount of preserved white matter available to support axonal conduction and neuroplasticity is substantially reduced, thereby limiting spontaneous neurological recovery. These biological mechanisms explain why intramedullary hemorrhage has consistently proven to be one of the most robust MRI biomarkers of poor neurological outcome across multiple clinical studies. This mechanistic understanding also reinforces the rationale for early neuroprotective interventions aimed at limiting secondary injury during the acute phase following SCI.

### Critical appraisal of current evidence

The growing body of MRI-based research has substantially improved our understanding of the anatomical substrates underlying traumatic SCI. Collectively, available studies consistently demonstrate that clinically complete SCI (AIS A) does not necessarily correspond to anatomical transection and that severe neurological deficits may arise from a spectrum of structural lesions, including edema, contusion, hemorrhage, and persistent compression. However, despite this broad conceptual agreement, the current evidence remains methodologically heterogeneous, limiting direct comparisons between studies and preventing the establishment of universally accepted MRI-based prognostic frameworks.

One of the major limitations of the existing literature lies in the heterogeneity of study design. Most MRI studies in acute SCI are retrospective, involve relatively small patient cohorts, and frequently originate from single specialized centers. Patient populations also differ substantially with respect to injury mechanism, neurological severity, spinal level, and timing of surgical decompression. Furthermore, MRI examinations are performed at variable time points after trauma, ranging from the first hours to several days after injury. Because spinal cord pathology evolves dynamically during the acute phase through edema progression, hemorrhagic expansion, inflammatory responses, and secondary ischemic injury, MRI findings obtained at different time points cannot always be directly compared. These methodological differences inevitably contribute to variability in reported prognostic performances.

An additional source of heterogeneity concerns MRI interpretation itself. Although several landmark studies have established the prognostic value of MRI, they rely on different lesion classification systems and imaging parameters. Kulkarni *et al.* initially described MRI abnormalities according to broad signal characteristics (4), whereas Ramón *et al.* proposed a three-pattern classification based on edema, contusion, and hemorrhage (5). Later studies by Miyanji and colleagues shifted toward quantitative MRI parameters, emphasizing lesion length, maximum canal compromise, maximum spinal cord compression, and the presence of intramedullary hemorrhage as independent predictors of neurological recovery (6). More recent investigations have combined qualitative and quantitative approaches, while others have focused primarily on lesion length or cord swelling. Consequently, identical pathological processes may be categorized differently depending on the study methodology, making direct comparisons challenging. Even commonly used terms such as *cord edema*, *contusion*, or *intramedullary hemorrhage* are not always defined according to identical radiological criteria, and standardized MRI descriptors are still lacking.

The prognostic significance of individual MRI biomarkers also deserves a more nuanced interpretation. Among currently available imaging findings, intramedullary hemorrhage appears to be the most reproducible predictor of poor neurological outcome across multiple studies. By contrast, the prognostic value of lesion length, cord swelling, edema extent, and maximum canal compromise remains more variable. While several authors reported strong correlations between increasing lesion length and poorer functional recovery, others observed weaker associations after adjustment for baseline neurological severity or surgical timing. These discrepancies likely reflect differences in imaging protocols, MRI timing, study populations, and statistical methodology rather than true biological contradictions. They also highlight the multifactorial nature of neurological recovery, which depends not only on anatomical injury severity but also on patient age, systemic trauma, early decompression, rehabilitation intensity, and secondary injury mechanisms.

These limitations underscore an important conceptual point: MRI should not be considered as a replacement for neurological examination but rather as a complementary source of biological information. The American Spinal Injury Association (ASIA) Impairment Scale remains the cornerstone of SCI assessment because it directly measures functional impairment and provides an internationally standardized language for clinical practice and research. However, neurological examination alone cannot characterize preserved tissue continuity, vascular disruption, intramedullary hemorrhage, or the spatial extent of structural damage. Conversely, MRI provides detailed anatomical information but cannot fully predict functional capacity or neuroplastic potential. A comprehensive evaluation of SCI therefore requires integration of both functional and structural assessments rather than reliance on either modality alone.

This integrated perspective is becoming increasingly relevant with the emergence of advanced MRI biomarkers. Conventional T2-weighted imaging already provides valuable prognostic information, but diffusion tensor imaging, quantitative susceptibility mapping, magnetization transfer imaging, myelin-sensitive sequences, functional MRI, and radiomic analyses may further refine lesion characterization in the coming years. Similarly, multimodal prognostic models combining neurological examination, structural imaging, electrophysiology, circulating biomarkers, and artificial intelligence-based image analysis may ultimately provide substantially greater diagnostic accuracy than any single parameter considered in isolation. Future prospective multicenter studies using standardized imaging acquisition protocols and centralized image interpretation will be essential to validate these approaches.

Finally, the concept of anatomically informed structural modifiers proposed in this review should be interpreted within this context. Although integrating lesion morphology into neurological assessment appears conceptually attractive and clinically intuitive, the currently available evidence does not justify the creation of a new MRI-based classification system. Rather, these structural modifiers should be regarded as a hypothesis-generating conceptual framework intended to stimulate discussion and future prospective validation. Their principal objective is not to replace the established ASIA Impairment Scale but to illustrate how structural imaging may complement functional assessment and contribute to a more biologically informed interpretation of traumatic SCI.

### Paradigm shift: toward anatomically informed assessment of traumatic SCI

The traditional dichotomous interpretation of traumatic SCI as complete versus incomplete has long structured clinical reasoning, prognostic assessment, and therapeutic expectations. While neurological classification using the AIS remains indispensable, this conceptual framework may oversimplify the complex anatomical and biological realities underlying traumatic SCI.

A clinically complete SCI does not invariably equate to complete anatomical destruction. MRI-based observations consistently demonstrate that severe neurological impairment may arise from heterogeneous lesion substrates, including edema, contusion, intramedullary hemorrhage, persistent compression, and, less frequently, complete anatomical transection, often with preserved tissue continuity. This distinction is not merely semantic but reflects fundamentally different pathological mechanisms, biological responses, and recovery potentials.

Revisiting the anatomical reality of neurologically complete injuries therefore encourages a more nuanced interpretation of injury severity. Rather than considering neurological completeness as a fixed endpoint, it may be more appropriately viewed as a functional state resulting from dynamic and evolving pathological processes. This perspective is consistent with the contemporary understanding of secondary injury cascades and the partial reversibility of certain tissue alterations during the acute phase following trauma.

The evolving understanding of SCI also highlights the limitations of purely functional classification systems when considered in isolation. While the AIS remains the cornerstone of neurological assessment, integrating structural lesion characterization provides an opportunity to refine the conceptual framework of injury severity without challenging the clinical relevance of existing classifications. MRI has consistently demonstrated that clinically complete SCI encompasses heterogeneous anatomical substrates ranging from potentially reversible edema to extensive intramedullary hemorrhage and, more rarely, complete anatomical transection. Consequently, neurological presentation alone cannot fully capture the biological complexity or regenerative potential of traumatic SCI.

In this context, we propose anatomically informed structural modifiers (Table 2) as a pragmatic conceptual framework designed to complement, rather than replace, neurological assessment. By incorporating lesion morphology and structural severity into clinical reasoning, this approach may improve prognostic stratification, facilitate therapeutic decision-making, and support more individualized patient counseling. Importantly, these structural modifiers should be regarded as a hypothesis-generating conceptual framework requiring prospective validation rather than as a new MRI-based classification system.

**Table 2:** The proposed structural modifiers conceptually map onto these MRI phenotypes but are not intended to constitute a new validated classification.

MRI phenotype	Main pathological substrate	Tissue continuity	Expected prognosis
Predominant edema	Reversible dysfunction	Preserved	Favorable
Contusion	Partial structural damage	Preserved	Intermediate
Hemorrhage	Vascular disruption	Variable	Poor
Transection	Anatomical disruption	Lost	Very poor

This paradigm shift carries important clinical implications. Prognostic models, therapeutic strategies, and rehabilitation pathways are likely to benefit from integrating functional and structural assessments rather than relying exclusively on neurological examination. Recognizing preserved tissue integrity may refine prognostic estimation, support individualized patient counseling, improve patient selection for neuroprotective and regenerative therapies, and contribute to a more biologically informed interpretation of traumatic SCI.

Future research should therefore focus on standardizing MRI descriptors, validating integrated clinico-radiological models in large prospective multicenter cohorts, and determining whether structural imaging biomarkers improve prognostic accuracy beyond neurological examination alone (16). Ultimately, traumatic SCI should be approached as a multidimensional disorder in which clinical presentation and anatomical reality coexist but do not always coincide. Bridging functional and anatomical perspectives represents a critical step toward precision medicine and more individualized management of traumatic SCI.

### Future directions: toward precision medicine in SCI

The progressive integration of advanced imaging into SCI assessment has the potential to fundamentally reshape the way injury severity is characterized. Rather than relying exclusively on neurological examination, future diagnostic strategies will likely combine functional evaluation with multimodal structural and biological biomarkers to generate a more comprehensive representation of spinal cord damage.

Conventional MRI already provides valuable prognostic information through the assessment of hemorrhage, edema, cord swelling, and lesion extent. However, emerging quantitative imaging techniques may substantially improve tissue characterization beyond what is currently achievable with routine clinical sequences. Diffusion tensor imaging (DTI) enables the evaluation of white matter integrity and axonal preservation, while myelin-sensitive techniques, quantitative susceptibility mapping, magnetization transfer imaging, and spinal cord functional MRI may provide complementary information regarding tissue viability, demyelination, microstructural organization, and residual functional connectivity. These imaging biomarkers have the potential to detect subtle structural preservation even in patients presenting with clinically complete neurological deficits.

Future prognostic models are also expected to become increasingly multimodal. Rather than depending on a single imaging parameter, prediction algorithms will likely integrate neurological examination, conventional MRI, quantitative MRI biomarkers, electrophysiological assessments, circulating biomarkers, and clinical variables into individualized prognostic models. Artificial intelligence and radiomics may further enhance this process by extracting imaging features that are not visually detectable and by identifying complex interactions between structural abnormalities and long-term neurological recovery. Such approaches may ultimately provide more accurate and reproducible prognostic estimates than current clinical classifications alone.

Improved anatomical characterization has important implications that extend beyond prognostic assessment. Patient selection remains one of the major challenges in neuroprotective and regenerative clinical trials, where considerable biological heterogeneity exists despite apparently similar neurological presentations. Better identification of preserved tissue bridges, hemorrhagic burden, or residual white matter integrity could facilitate patient stratification according to biological recovery potential, improve interpretation of therapeutic efficacy, and optimize individualized treatment strategies. This concept is particularly relevant for emerging regenerative approaches, including cell-based therapies, biomaterials, and neurorestorative interventions, where lesion morphology is likely to influence treatment response.

Beyond clinical trials, these advances may also directly improve routine patient management. More accurate lesion characterization could facilitate individualized prognostic counseling, optimize surgical decision-making, guide rehabilitation planning, and help establish realistic functional expectations for patients and their families. Importantly, recognizing that neurologically complete injuries do not necessarily imply complete anatomical destruction may reduce overly deterministic prognostic assumptions during the acute phase and support a more personalized therapeutic strategy. MRI-derived lesion characterization may also assist clinicians in identifying patients who could benefit from aggressive neuroprotective strategies, early surgical decompression, or enrollment in regenerative medicine trials.

Nevertheless, several challenges remain before such approaches can be widely implemented. Prospective multicenter studies using standardized MRI acquisition protocols, centralized image analysis, and harmonized imaging terminology will be essential to validate imaging biomarkers and establish clinically applicable prognostic algorithms. Future research should also determine whether anatomically informed assessment improves clinical decision-making and patient outcomes beyond existing neurological classifications.

Ultimately, the future of SCI assessment is likely to rely on a multidimensional precision medicine approach in which neurological examination, advanced imaging, electrophysiology, biological biomarkers, and computational modeling are integrated to characterize each patient’s unique injury profile. Such an approach has the potential to improve diagnostic reliability, refine prognostic accuracy, optimize patient selection for emerging therapies, and ultimately facilitate more individualized management of traumatic SCI. Future SCI research should therefore move beyond the traditional binary distinction between complete and incomplete injuries toward integrated clinico-radiological models capable of capturing the biological heterogeneity of traumatic SCI. Future regenerative clinical trials should increasingly integrate MRI-based structural biomarkers as stratification variables in order to reduce biological heterogeneity and improve interpretation of treatment effects.

## Conclusion

Traumatic SCI remains a highly complex condition in which clinical presentation and underlying anatomical damage do not always coincide. Although neurological classification using the AIS is fundamental to patient evaluation, the concept of clinical completeness should not be interpreted as a direct surrogate of anatomical destruction.

MRI has demonstrated that clinically complete SCI frequently encompasses heterogeneous lesion substrates, including edema, contusion, hemorrhage, and compression, often with preserved tissue continuity. This clinico-anatomical dissociation reflects the dynamic and multifactorial nature of spinal cord pathology, shaped by both primary mechanical insult and secondary injury mechanisms.

Recognizing this complexity refines prognostic interpretation and supports a more comprehensive assessment of injury severity. Integrating neurological examination with structural imaging improves clinical decision-making, informs therapeutic strategies, and facilitates more accurate patient counseling. In this perspective, anatomically informed structural modifiers may represent a pragmatic conceptual framework to complement functional classification and better capture lesion heterogeneity.

Ultimately, recognizing that neurologically complete injuries are not necessarily anatomically complete represents a fundamental paradigm shift toward precision medicine in traumatic SCI. This anatomical reality should be systematically considered when interpreting clinically complete SCI. Future therapeutic strategies should increasingly target biological lesion phenotypes rather than neurological categories alone. Traumatic SCI should no longer be viewed as a binary condition but rather as a biological continuum in which functional impairment and structural damage only partially overlap.
